# Supplementary material for: Isolation and Characterization of the First Zobellviridae Family Bacteriophage Infecting Klebsiella pneumoniae
Source: Int J Mol Sci. 2023 Feb 17;24(4):4038. doi: 10.3390/ijms24044038 (PMC9960094; doi:10.3390/ijms24044038)
Supplement: Supplementary file 1 [file ijms-24-04038-s001.zip › Supplementary Figure S4.pdf]

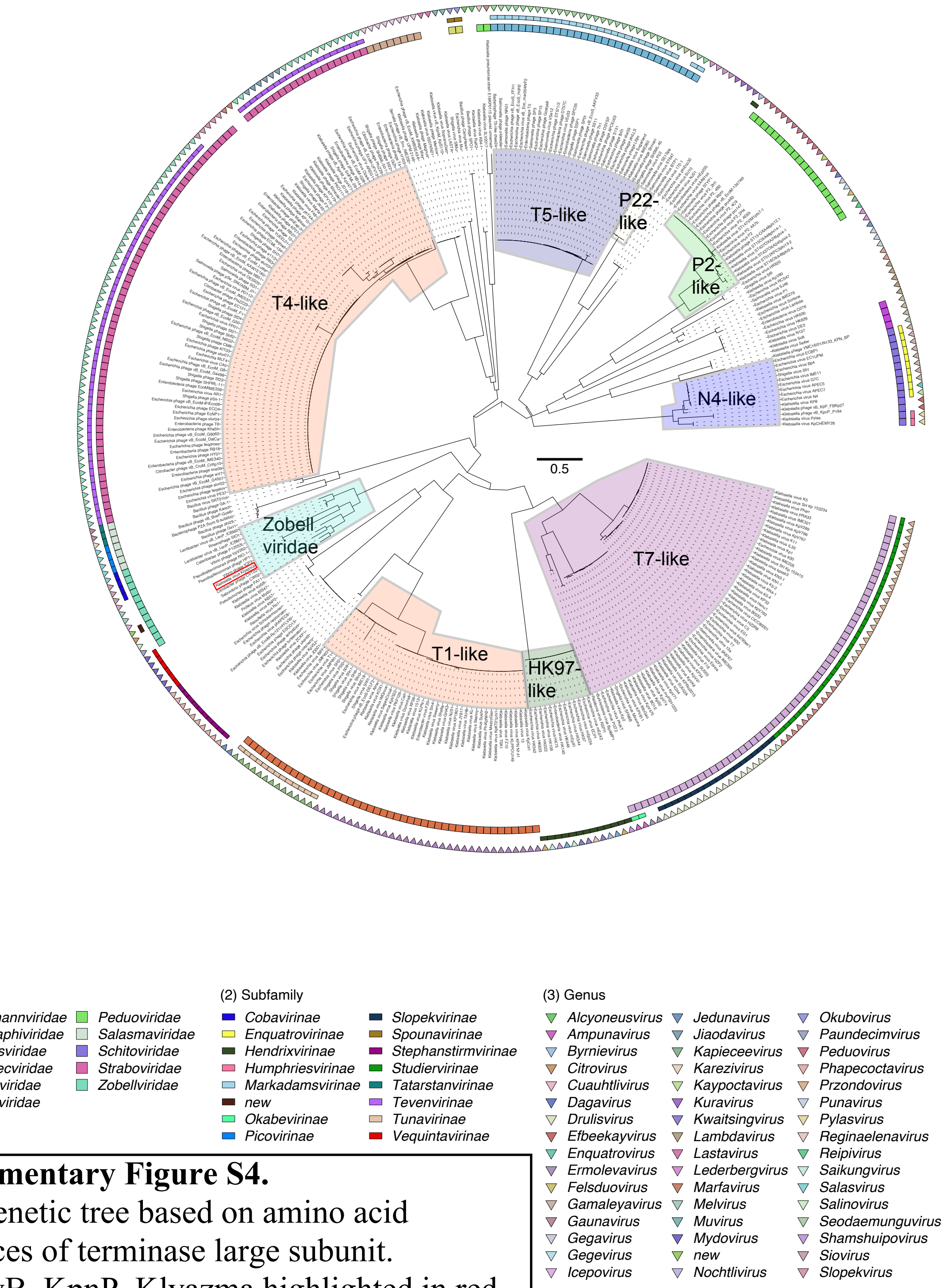

Supplementary Figure S4.
Phylogenetic tree based on amino acid sequences of terminase large subunit. Phage vB_KpnP_Klyazma highlighted in red.
