# Supplementary material for: Isolation and Characterization of the First Zobellviridae Family Bacteriophage Infecting Klebsiella pneumoniae
Source: Int J Mol Sci. 2023 Feb 17;24(4):4038. doi: 10.3390/ijms24044038 (PMC9960094; doi:10.3390/ijms24044038)
Supplement: Supplementary file 1 [file ijms-24-04038-s001.zip › Supplementary Figures.pdf]

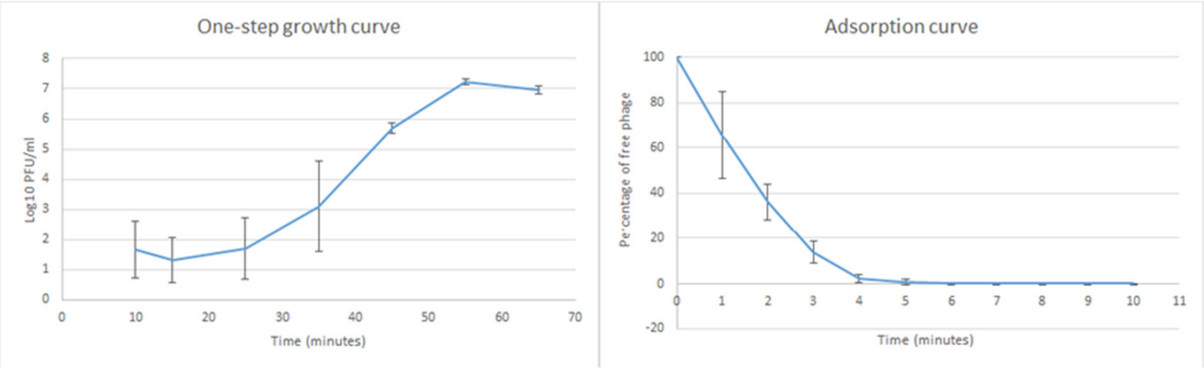

Supplementary Figure S1. One-step growth curve (A) and adsorption curve (B) of bacteriophages vB_KpnP_Klyazma.

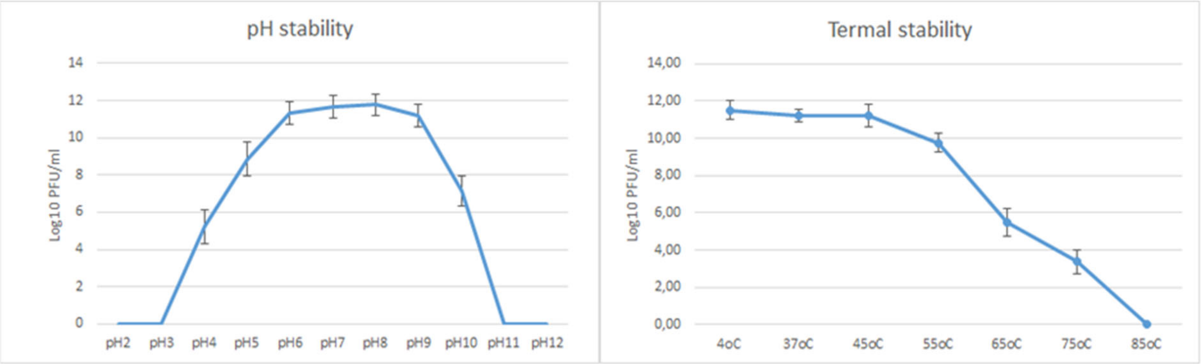

Supplementary Figure S2. pH tolerance (A) and thermal tolerance (B) of bacteriophage vB_KpnP_Klyazma.

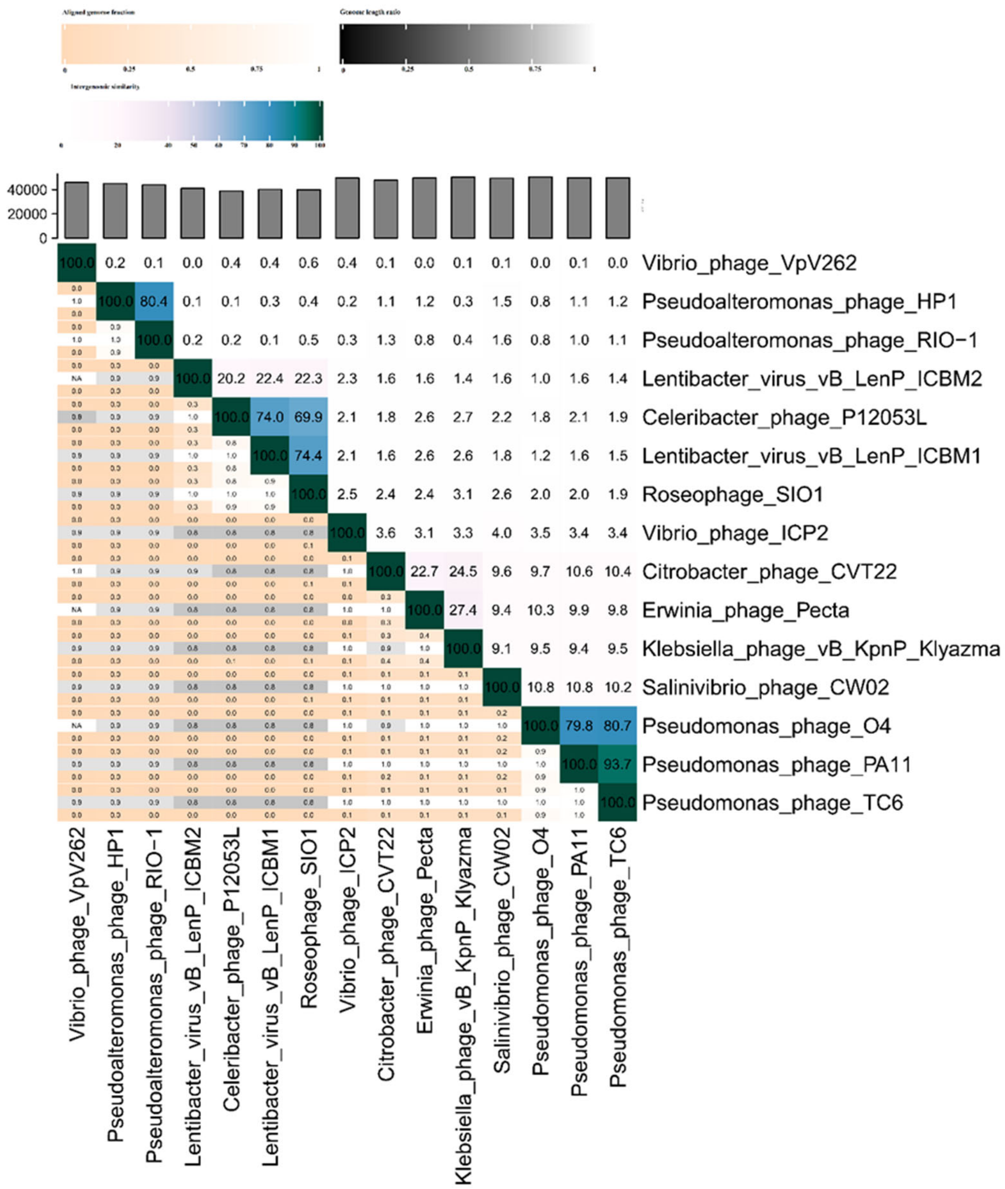

Supplementary Figure S3. Nucleotide based, intergenomic similarities between the members of the here newly proposed *Zobellviridae* family. The intergenomic similarities were calculated using the VIRIDIC webservice (viridic.icbm.de).

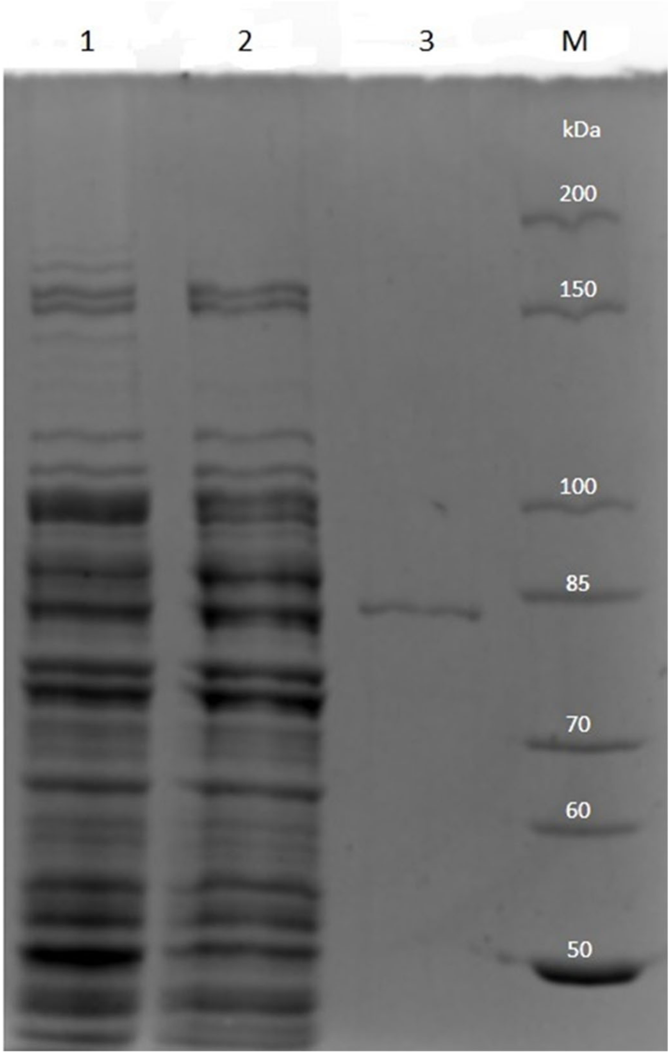

Supplementary Figure S5. SDS-PAGE showing induced expression and purification of the Kl-dep depolymerase. SDS-PAGE was performed with 10% polyacrylamide gels, 80 V for 30 min, 120-200V for an hour. Lane 1, proteins profile of *E. coli* T7 Express *lysY/I*^{q} cells after 18 h induction; lane 2, supernatant of the cells lysate; lane 3, purified protein; lane 4, marker.
